# Comparison of five different fluoroscopic methods for identifying the MPFL femoral footprint

**DOI:** 10.1007/s00402-024-05213-9

**Published:** 2024-02-24

**Authors:** Tuluhan Yunus Emre, Hakan Cetin, Huseyin Selcuk, Koray Kaya Kilic, Faruk Aykanat, Levent Sarikcioglu, Ozkan Kose

**Affiliations:** 1 Department of Orthopaedics and Traumatology, Kadikoy Hospital, Acıbadem University, Istanbul, Turkey; 2 Department of Orthopedics and Traumatology, Antalya Training and Research Hospital, Varlık mah., Kazım Karabekir cd., Muratpasa, 07100 Antalya, Turkey; 3 Department of Radiology, Antalya Training and Research Hospital, Antalya, Turkey; 4 Medical Faculty Department of Orthopaedics and Traumatology, Sanko University, Gaziantep, Turkey; 5 Department of Anatomy, Medical Faculty, Akdeniz University, Antalya, Turkey

**Keywords:** Medial patellofemoral ligament, Patellofemoral instability, Patellar dislocation, Fluoroscopy, Femoral tunnel, Schottle point

## Abstract

**Purpose:**

The success of medial patellofemoral ligament (MPFL) reconstruction is closely linked to the precise positioning of the femoral tunnel. Intraoperative fluoroscopy is commonly utilized to identify the MPFL footprint. This study aimed to ascertain the most accurate fluoroscopic method among the five previously described methods used to determine the MPFL femoral footprint.

**Materials and methods:**

Using 44 well-preserved dry femur bones, the MPFL femoral insertion site was demarcated using anatomical bony landmarks, namely the center of the saddle sulcus between the medial epicondyle, adductor tubercle and gastrocnemius tubercle. Fluoroscopic true lateral knee images were acquired and measurements taken, referencing established methods by Schottle et al., Redfern et al., Wijdicks et al., Barnett et al., and Kaipel et al. The distance between anatomic and fluoroscopic MPFL footprints was then measured on digital fluoroscopic images. The accuracy of the locations was compared using a margin of error of 5 and 7 mm.

**Results:**

The Schottle method consistently emerged superior, showcasing the smallest mean distance (3.2 ± 1.2 mm) between the anatomic and radiographic MPFL footprints and a high in-point detection rate of 90.9% under 5 mm criteria. While the Redfern method displayed perfect accuracy (100%) within the 7 mm criteria, the Schottle method also performed 97.7% accuracy.

**Conclusions:**

For intraoperative identification of the MPFL footprint using fluoroscopy, the Schottle method is the most consistent and accurate among the assessed methods. Thus, its accuracy in detecting the MPFL footprint makes it recommended for MPFLR to ensure optimal outcomes.

**Level of evidence:**

Level IV, cadaveric study.

## Introduction

The medial patellofemoral ligament (MPFL) is often injured in acute lateral patellar dislocations. Kluczynski et al. analyzed 35 research studies involving 2558 patients with acute patellar dislocations and found that nearly 95% had injuries to the MPFL [1]. The MPFL, as the main stabilizer of the patella against lateralizing forces between 0 and 30° of knee flexion, is primarily responsible for preventing lateral patellar dislocations, contributing to over half of the restraining force according to previous biomechanical studies. [2–4]. While first-time dislocations can be managed conservatively, recurrent dislocations usually require surgical intervention [5, 6]. MPFL reconstruction (MPFLR) is the mainstay of surgical treatment for recurrent patellar dislocation, which corrects the ligament deficiency and stabilizes the patella. The success of MPFLR is closely linked to the precise positioning of the femoral tunnel. Recent biomechanical studies underscore the critical role of correct femoral tunnel placement in maintaining normal patellofemoral kinematics [7, 8]. Furthermore, clinical studies have consistently shown that correct femoral tunnel placement is associated with fewer complications and better outcomes [9–11].

There are two primary intraoperative techniques for determining the MPFL femoral footprint: the open and the fluoroscopic methods. The open method identifies the MPFL femoral footprint by surgically dissecting and palpating specific anatomical landmarks, such as the adductor tubercle, gastrocnemius tubercle, medial epicondyle, and saddle sulcus. While it provides high accuracy without radiation exposure, it requires extensive dissection, resulting in significant scarring. Furthermore, it requires considerable experience [12–15]. The fluoroscopic method, on the other hand, relies on radiographic landmarks on a true lateral knee image to locate the MPFL femoral footprint. It is minimally invasive and can be performed through an aesthetically acceptable small incision [16]. However, this technique is affected by even minor errors in obtaining the true lateral knee radiograph [17].

**Fig. 1:**
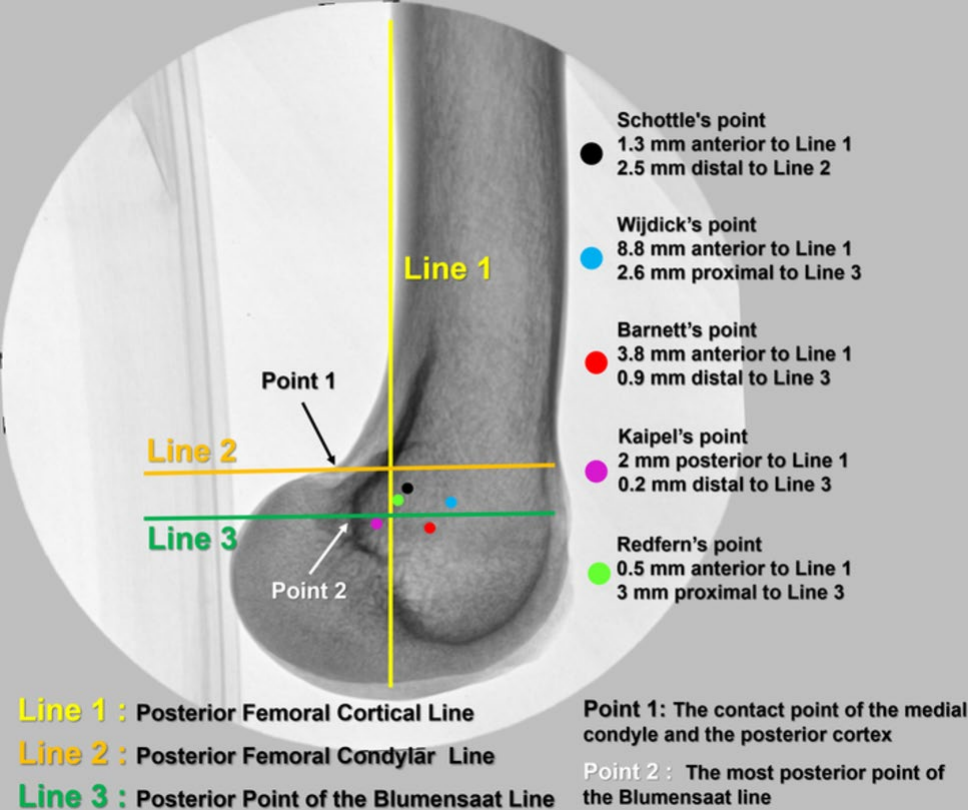
Schematic representation of fluoroscopic methods to identify the femoral footprint of the MPFL. Posterior femoral cortical line (line 1, yellow line), posterior femoral condylar line (line 2, orange line), and posterior Blumensaat line (line 3, green line). The black, blue, red, purple, and green dots indicate the distance and location of the fluoroscopy methods from line 1, line 2, and line 3, respectively, on the true lateral knee fluoroscopic image

Schöttle et al. first described the fluoroscopic technique in 2007 [16]. Subsequently, it gained widespread acceptance and was implemented in practice. Schöttle et al. dissected eight cadavers to identify the MPFL, positioned a metal indicator at its femoral attachment site, and standardized the projection of this point on a true lateral knee fluoroscopic image. According to Schöttle et al., the MPFL footprint was situated 1.3 mm anterior to the posterior cortical extension, 2.5 mm distal to a perpendicular line intersecting the origin of the posterior medial femoral condyle, and 3 mm proximal to a perpendicular line intersecting the posterior point of the Blumensaat line. Nonetheless, subsequent researchers have proposed that this methodology might lack precision and have advocated for alternative fluoroscopic techniques [18–21] (Fig. 1). To date, no research in the existing literature has evaluated the accuracy of these methods compared to one another. This study aims to ascertain the most accurate method among the five techniques to pinpoint the MPFL footprint on intraoperative fluoroscopy.

## Materials and methods

### Study design and specimens

A total of 44 well-preserved dry femur bones were selected from a collection of 142 dry femur bones housed in the Akdeniz University Clinical Anatomy Laboratory and were included in this study. Bones with unclear anatomical landmarks and broken or damaged bones were excluded from the study. None of the bones included in the study were found to have trochlear dysplasia through macroscopic examination. The sex and age at the time of death of the dry femur bones were unknown. The local ethics committee approved the study protocol (20.07.2023-10/10).

### Determination of MPFL footprint on dry bones

**Fig. 2:**
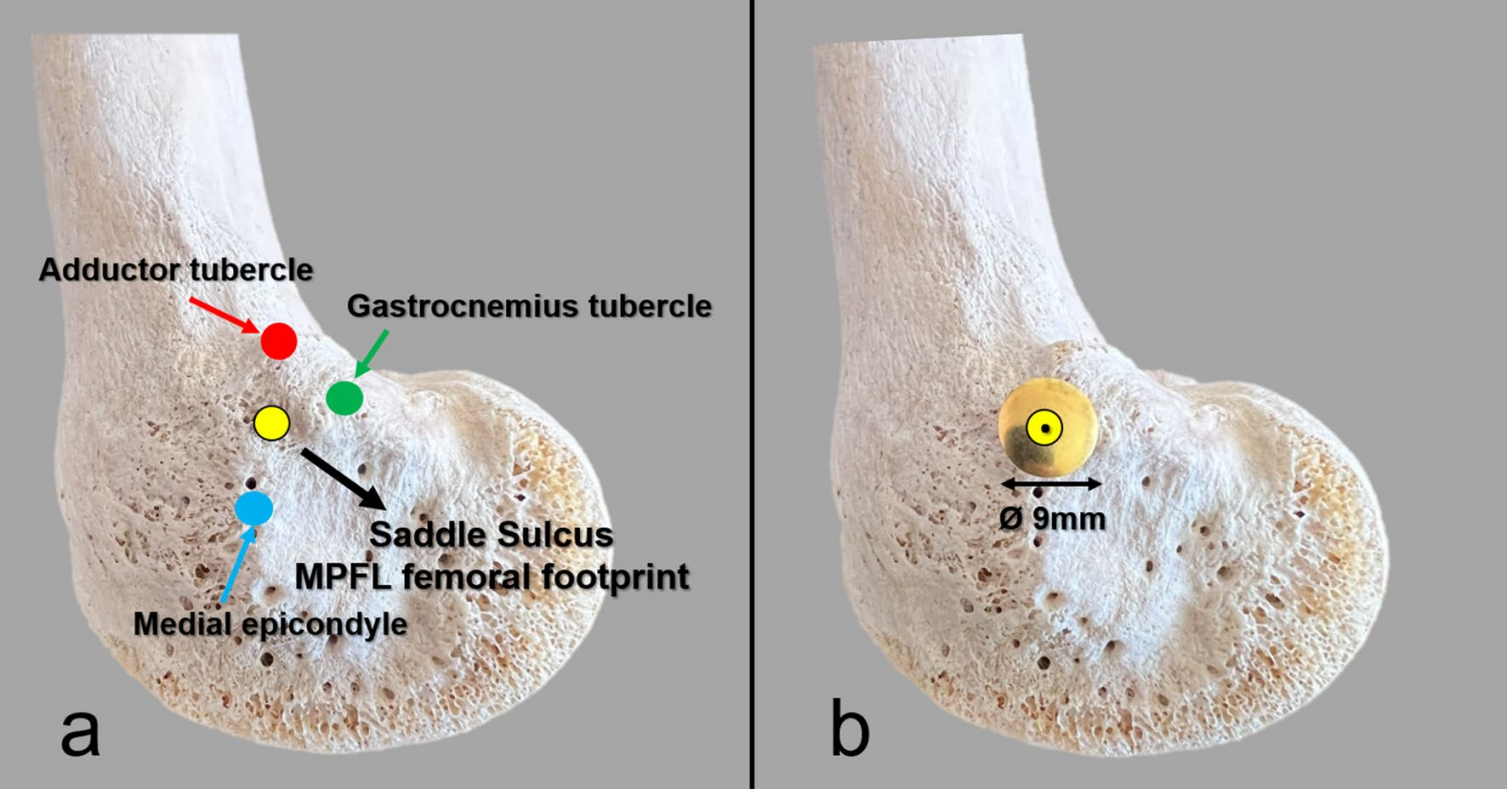
**a** The anatomic landmarks used to identify the MPFL femoral attachment. **b** Appearance of the metal thumbtack with a head diameter of 9 mm

A committee consisting of an anatomist and two senior orthopedic surgeons examined all bones and collaboratively identified the femoral MPFL insertion site using bony landmarks. The groove called the saddle sulcus between the adductor tubercle, gastrocnemius tubercle, and medial epicondyle reported in previous anatomic studies was accepted as the MPFL attachment site, and its center was marked with a metal 9 mm thumbtack (Fig. 2) [22, 23].

### Fluoroscopic imaging

**Fig. 3:**
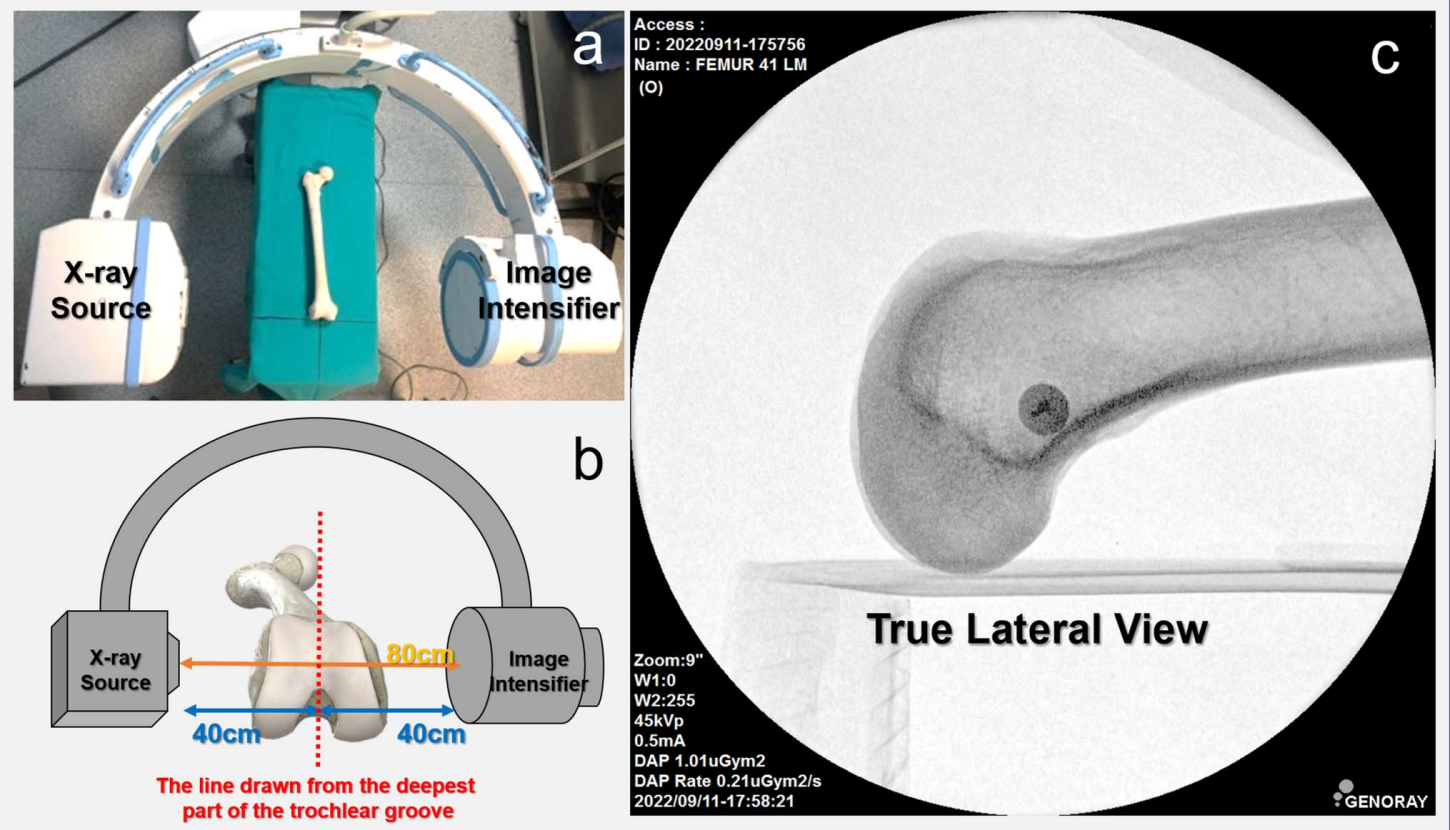
**a** The position of the femur and the fluoroscopic set-up. **b** The dry femur specimen was positioned at the midpoint of the distance between the X-ray source and the image intensifier. **c** A true lateral knee fluoroscopic image was obtained

Fluoroscopic imaging of the specimens was performed using c-arm fluoroscopy (Genoray, Fluoroscopic X-ray System, Model: Zen-500, Gyeonggi-do, Korea). The distance between the X-ray source and the image intensifier was 80 cm. The deepest part of the trochlea aligned at the midpoint of this distance and the thumbtack was centralized to the image intensifier. Standard true lateral knee images were obtained while the X-ray source was on the lateral side. The shots were taken with a range of 45–52 kVp and 0.4–1.8 mA, respectively. The fluoroscopy set-up is shown in Fig. 3. To obtain an image with the exact overlap of the femoral condyles, 5–10 images were acquired for each femur, and the most precise image was selected for the study. On a true lateral femur radiograph, the posterior femoral condyles should overlap and appear as a single condyle. All images with a double contour appearance were excluded.

### Radiographic measurements

Digital images were saved in DICOM format and imported into RadiAnt DICOM Viewer software (Medixant Ltd., Poland, V.2023.1). Three reference lines were drawn on the digital images as described by Schöttle et al.: posterior femoral cortical line (Line 1), posterior femoral condylar line (Line 2), and most posterior of the Blumensaat line (Line 3) (Fig. 1). The fluoroscopic MPFL footprint was then marked using the distances reported by Schöttle et al. [16], Redfern et al. [18], Wijdicks et al. [20], Barnett et al. [19], and Kaipel et al. [21]. The distance between the center of the metal thumbtack, the anatomic MPFL point, and these points was measured. Since the head of the metal pin is known to be 9 mm, the measured distances were corrected accordingly to calibrate the magnification.

In addition, a coordinate system was established using the same radiographic reference lines to understand the direction of the deviation. The coordinates of the fluoroscopic MPFL point were set as the origin (*x*: 0, *y*: 0), and the coordinates of the anatomic MPFL pins were determined and plotted on the coordinate grid. This enabled evaluation of both the extent and the spatial direction of the deviation. Two criteria were set to measure the accuracy in locating the MPFL anatomical footprint. Schöttle et al. reported that the anatomic MPFL footprint was within a 5 mm diameter circle on their radiologic method and suggested that this distance did not negatively disrupt the isometry of the ligament in the light of previous biomechanical studies [16]. However, Servien et al. later revised this to a 7 mm diameter, aligning with the standard 7 mm drill used for creating the femoral tunnel, and considered a deviation of up to 7 mm as acceptable [24]. Thus, variations exceeding 5 mm (Criterion 1) and 7 mm (Criterion 2) from the midpoint of the radiographic compared to the anatomical MPFL imprints were deemed to be outside the normal range [16, 24].

Two independent observers who were orthopedic surgeons performed the measurements once. Observers were blinded to their own and the other observer's measurements. Interobserver reliability was tested using the interclass correlation coefficient (ICC). ICC values were scored according to the following criteria: an ICC value below 0.5 indicates poor reliability, between 0.5 and 0.75 indicates moderate reliability, between 0.75 and 0.9 indicates good reliability, and above 0.9 indicates excellent reliability [25]. Interobserver reliability was good and excellent for all measurements; thus, the mean of observers’ measurements was used for the final analysis (Table 1).

**Table 1:** Interobserver reliability of distance measurements between anatomic and radiographic MPFL footprint

Fluoroscopic methods	ICC (95% CI)	Interpretation
Redfern et al. (mm ± SD)	0.868 (0.771–0.926)	Good
Barnett et al. (mm ± SD)	0.893 (0.813–0.940)	Good
Kaipel et al. (mm ± SD)	0.823 (0.699–0.900)	Good
Wijdicks et al. (mm ± SD)	0.947 (0.905–0.971)	Excellent
Schottle et al. (mm ± SD)	0.901 (0.826–0.945)	Excellent

*SD* standard deviation, *ICC* interclass correlation coefficient, *CI* confidence interval

### Statistical analysis

Statistical evaluations were conducted using SPSS Statistics Base v.23 on Windows. Continuous data were characterized by mean, standard deviation, and range, whereas categorical data were represented in frequencies and percentages. The normality of continuous variables was ascertained using the Kolmogorov–Smirnov test. Parametric tests were implemented for data following a normal distribution, and nonparametric tests were chosen for those not fitting this distribution. The paired sample *t* test was adopted to contrast continuous variables, and the chi-squared test was designated for comparisons of categorical data. A *p* value less than 0.05 was considered to indicate statistical significance.

## Results

### Comparison of distance between anatomic and fluoroscopic MPFL points

**Fig. 4:**
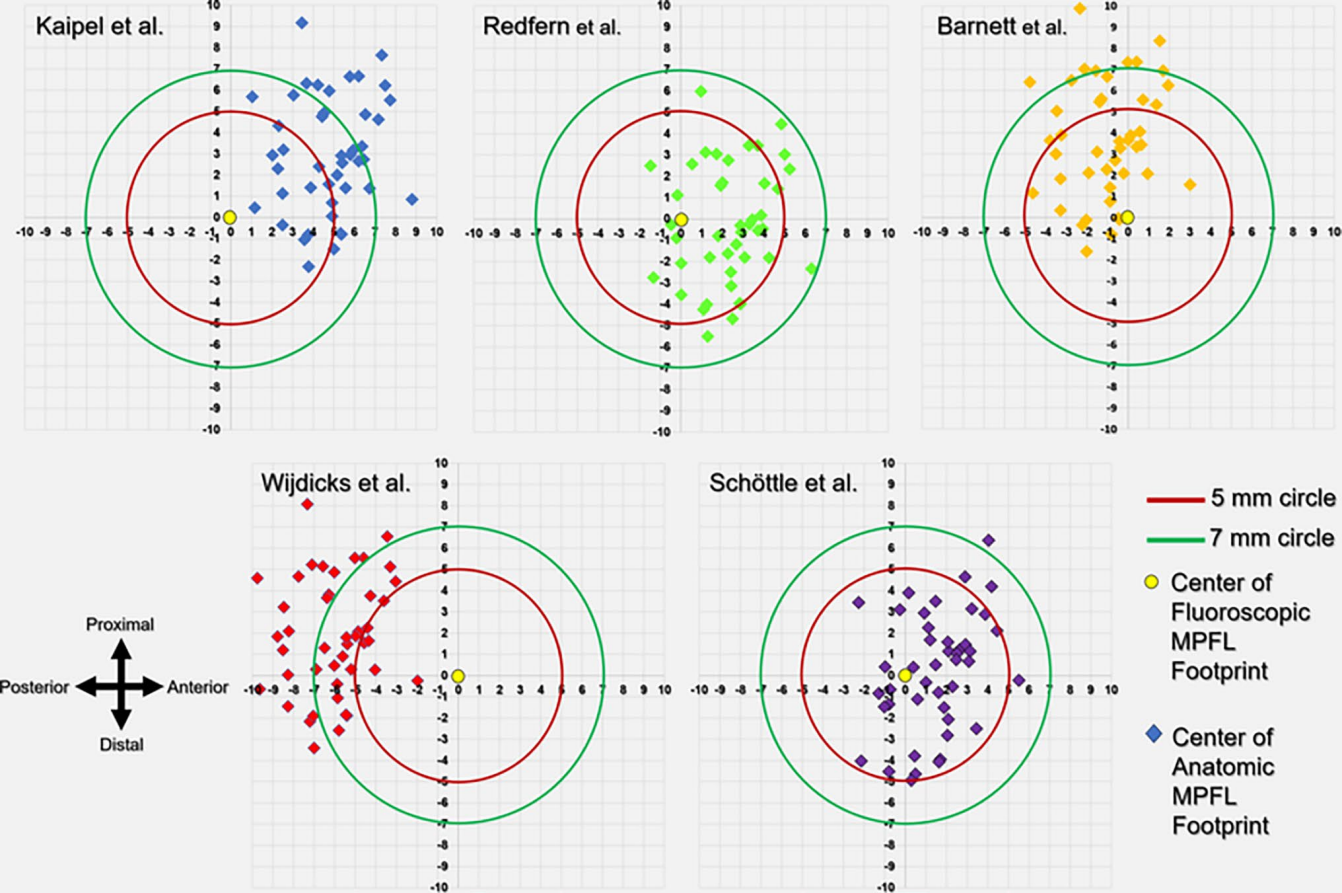
The distribution of fluoroscopic MPFL points in accordance to anatomic MPFL point (yellow dot). The coordinates of the anatomic MPFL point is (0, 0)

Table 2 delineates the comparative analysis of distances between the anatomic and the fluoroscopic MPFL points as determined by five methods. The Schöttle method demonstrated the most proximate mean distance of 3.2 ± 1.2 mm. In contrast, the Wijdicks method manifested the most disparate mean distance, registering at 6.8 ± 1.8 mm. Utilizing a one-way ANOVA, a statistically significant variation in distances was identified across the techniques (*p* = 0.001). Further post hoc comparisons using the Tukey test revealed several pairs of methods with significant differences. Although the Schöttle method was similar to the Redfern method, it significantly differed from the other three methods. Figure 4 shows the accuracy of the methods on the coordinate system.

**Table 2:** Comparison of distance between the anatomic MPFL and the fluoroscopic MPFL points according to the methods

Fluoroscopic methods		Distance (mm ± SD)	*p* value
1	Redfern et al.	3.6 ± 1.5	**0.001**^1^
2	Barnett et al.	4.4 ± 2.3	
3	Kaipel et al.	6.1 ± 2.1	
4	Widjick et al.	6.8 ± 1.8	
5	Schottle et al.	3.2 ± 1.2	

Post hoc multiple comparisons			
Pairs	*p* value	Pairs	*p* value
1 vs. 2	0.330^2^	2 vs. 4	**0.001**^2^
1 vs. 3	**0.001**^2^	2 vs. 5	0.033^2^
1 vs. 4	**0.001**^2^	3 vs. 4	0.395^2^
1 vs. 5	0.845^2^	3 vs. 5	**0.001**^2^
2 vs. 3	**0.001**^2^	4 vs. 5	**0.001**^2^

^1^ANOVA, ^2^Tukey test. *p* > 0.005 is significant. Bold *p* values are significant

### Anatomic MPFL point detection rates: a comparative analysis based on 5 mm and 7 mm criteria

Table 3 presents the MPFL femoral tunnel placement accuracy within the acceptance limits of 5 and 7 mm. Under the stringent 5 mm criteria, the Schöttle method emerged as the most precise, boasting an impressive in-point detection rate of 90.9%. In stark contrast, the Wijdicks method exhibited the least precision, with an in-point detection rate of 11.4%. A chi-squared test confirmed the presence of a significant statistical difference in the detection rates among the methods (*p* = 0.001). Further analysis through post hoc multiple comparisons highlighted pronounced differences between several method pairings. Similar to distance measurements, the Schöttle method was similar to the Redfern method but significantly better than the others. In the context of the 7 mm criteria, the Redfern technique demonstrated unparalleled precision, achieving an impeccable in-point detection rate of 100%.

**Table 3:** Comparison of MPFL femoral tunnel placement accuracy within 5 and 7 mm acceptance limits

Variables	5 mm criteria		*p* value
	Out *n* (%)	In *n* (%)	
1. Redfern et al.	8 (18.2%)	36 (81.8%)	0.001^1^
2. Barnett et al.	18 (40.2%)	26 (59.1%)	
3. Kaipel et al.	30 (68.2%)	14 (31.8%)	
4. Wijdicks et al.	39 (88.6%)	5 (11.4%)	
5. Schottle et al.	4 (9.1%)	40 (90.9%)	

Post hoc multiple comparisons			
Pairs	*p* value	Pairs	*p* value
1 vs. 2	0.052	2 vs. 4	**< 0.000**
1 vs. 3	**< 0.000**	2 vs. 5	**0.001**
1 vs. 4	**< 0.000**	3 vs. 4	0.036
1 vs. 5	0.352	3 vs. 5	**< 0.000**
2 vs. 3	0.018	4 vs. 5	**< 0.000**

	7 mm criteria		*p* value
	Out *n* (%)	In *n* (%)	
1. Redfern et al.	0 (0%)	44 (100%)	0.001^1^
2. Barnett et al.	9 (20.5%)	35 (79.5%)	
3. Kaipel et al.	14 (31.8%)	30 (68.2%)	
4. Wijdicks et al.	21(47.7%)	23 (52.3%)	
5. Schottle et al.	1 (2.3%)	43 (97.7%)	

Post hoc multiple comparisons			
Pairs	*p* value	Pairs	*p* value
1 vs. 2	**0.004**	2 vs. 4	0.012
1 vs. 3	**< 0.000**	2 vs. 5	0.014
1 vs. 4	**< 0.000**	3 vs. 4	0.190
1 vs. 5	1.000	3 vs. 5	**< 0.000**
2 vs. 3	0.331	4 vs. 5	**< 0.000**

Out indicates the outside of the 5 mm circle, and In indicates the inside of the 5 mm circle

^a^Chi-squared test. ^2^Bonferroni correction *p* > 0.005 is significant. Bold *p* values are significant. Numbers in the pairs column designate the fluoroscopic methods

Conversely, the Wijdicks technique revealed the highest propensity for error, with an out-point detection rate of 47.7%. A subsequent chi-squared test validated the significant variability in detection rates among the techniques (*p* = 0.0011). Further in-depth post hoc analysis illuminated the Redfern method's discernible differences when contrasted against the Barnett, Kaipel, and Wijdicks methods but not the Schöttle method.

## Discussion

Successful MPFLR requires careful positioning of the femoral tunnel to restore normal patellofemoral kinematics and minimize postoperative complications. However, a key challenge is the accurate intraoperative identification of the MPFL femoral footprint, a prerequisite for optimal outcomes. This study aimed to determine the most accurate of the five fluoroscopic methods used for intraoperative identification of the MPFL footprint. Our results demonstrated the superiority of the Schöttle method in accurately identifying the anatomic MPFL point. While the Redfern method excelled in the 7 mm criteria with perfect accuracy (100%), the Schöttle method appears to be the most consistent across the board, having the smallest distance in the MPFL point comparison and the highest accuracy rate in the 5 mm criteria. It also performed accurately (97.7%) in the 7 mm criteria. Therefore, based on the combined evaluation of distance and detection rate accuracy, the Schöttle method can be considered the best overall among the tested methods.

There may be several reasons for the variation in recommendations for fluoroscopic detection of the MPFL footprint in the relevant literature. However, the most significant reason for the diversity is the individual variations in knee anatomy. As with all anatomical structures, the femoral attachment site of the MPFL is subject to variations in location and shape. These findings have been demonstrated in many previous anatomic studies. In their systematic review, Aframian et al. reviewed a total of 67 anatomic studies and reported that 16 different locations of the MPFL femoral attachment site were defined [26]. In another systematic review, Placella et al. reviewed 13 anatomic studies, including 312 knees, and found that the femoral insertion was at the adductor tubercle in 29.6% of cases, the medial epicondyle of the femur in 17.8%, and other sites in the remaining 44% [27]. In addition to location, the shape of the MPFL footprint and adjacent anatomic landmarks are variable. Dandu et al. investigated the topography of landmarks used in MPFLR and found significant variability in their morphology and spatial relationship to the MPFL footprint. The medial epicondyle (ME) showed significantly greater variance in volume compared to the adductor tubercle (AT) and gastrocnemius tubercle (GT), which were more consistent in morphology [28]. These findings suggest that the femoral attachment site of the MPFL is subject to frequent anatomical variation. Defining an anatomical structure with a single constant point is challenging given the diverse range of anatomical variations.

The second reason could be the methodological differences between the studies, mainly regarding sample preparation and imaging. The MPFL is not a true ligament but a functional layer of the medial retinaculum [29]. The dissection of these structures is complex, and unfortunately, several studies do not provide details of the dissection. Some authors in earlier studies could not even clearly identify the MPFL in their dissections [2, 30]. Again, radiographic imaging is subject to several intrinsic errors. It is difficult to obtain a true lateral knee radiograph. In our study, a minimum of 5–10 images were taken to obtain perfect condylar overlap. Balcerek et al. studied how minor deviations from a true lateral fluoroscopic view impact the accuracy of femoral tunnel placement. They showed that even slight deviations (2.5°–5°) in positioning led to significant shifts in the femoral MPFL insertion point, emphasizing the critical need for precision in achieving a true lateral view during surgery [17]. In addition, magnification and calibration errors can alter the result in an area where distance measurements are minute. For example, the point described by Redfern et al. is only 0.5 mm from the posterior cortical line [18]. Wijdicks et al. criticized Schottle’s methodology for not using proper specimen calibration and not performing reliability between examiners to validate their measurements [20]. Distance measurement is also affected by bone size. A 13-year-old girl and a 17-year-old boy will have different knee sizes and, therefore, different distances to the landmarks. The reliability of the measurements is another source of error [31]. Identifying guide points and drawing guidelines are subjective and thus prone to interobserver variations. Finally, almost all these studies were performed on a limited number of cadavers without apparent abnormality. On the other hand, the accuracy of fluoroscopy-guided tunnel placement, particularly in knees with severe trochlear dysplasia (Types C and D), is markedly decreased compared to mild trochlear dysplasia [32].

Based on these challenges, several authors advocated individualized detection of MPFL footprint [22, 33, 34]. Previous anatomical dissection studies have shown that the groove between the AT, GT, and medial epicondyle, also known as the saddle sulcus, is a constant area for the MPFL footprint, and this area can be visualized or palpated through an open dissection [22, 23, 35–38]. Although Zang et al. [15] supported the sulcus localization technique as a reliable and accurate method for MPFL femoral tunnel positioning, Abreu-E-Silva et al. [39] highlighted significant inaccuracies with the palpation method, even when performed by experienced surgeons. Similarly, Koh and Zimmerman reported a palpation error rate of approximately 20% [40]. A recent systematic study by Heindel et al. comparing open and fluoroscopic techniques showed no significant difference in complications or outcomes [41]. Nonetheless, it is crucial to acknowledge that in particular cases, especially during revision surgeries, detecting anatomical landmarks may prove problematic due to altered anatomy. In such instances, reliance on fluoroscopic guidance becomes vital and potentially the sole viable option.

The present study has several notable strengths and inherent limitations. The primary limitation arises from using dry femoral bone devoid of soft tissues. Thus, the identification of the MPFL footprint was based on bone landmarks rather than the ligament itself. The variability between the bone landmarks and the MPFL footprint introduces a potential bias. However, in many previous anatomical and radiological studies, the saddle sulcus has been identified and widely accepted as the site of MPFL attachment [22, 23, 35–38]. There was no trochlear dysplasia in the femurs included in the study. Considering that almost all patients undergoing MPFLR have varying degrees of trochlear dysplasia, the findings may not be generalizable to patients with patellofemoral instability. However, nearly all studies, including Schöttle et al., were performed on normal knees. Despite a rigorous methodological approach, errors in acquiring fluoroscopic images and measurements cannot be excluded. Multiple fluoroscopic images were obtained to minimize these errors, and the best true lateral knee radiograph was selected. In addition, measurements were made by different observers and used after being found reliable. Length measurements were also calibrated to eliminate magnification effects.

## Conclusions

In conclusion, the comprehensive analysis of this study highlights the Schöttle method as the most accurate and consistent technique for identifying the MPFL femoral footprint using intraoperative fluoroscopy. With the smallest mean distance between the anatomic and radiographic MPFL footprints and a high in-point detection rate, it emerges as the superior approach among the five methods evaluated. Future research should focus on refining these fluoroscopic techniques to cater to the individual variations in knee anatomy, particularly in patients with severe trochlear dysplasia.

## Data Availability

Data are available from the authors upon reasonable request.

## Abbreviations

MPFL: Medial patellofemoral ligament
MPFLR: Medial patellofemoral ligament reconstruction
TT–TG: Tibial tubercle–trochlear groove

## References

[1] Kluczynski MA, Miranda L, Marzo JM (2020). Prevalence and site of medial patellofemoral ligament injuries in patients with acute lateral patellar dislocations: a systematic review and meta-analysis. Orthop J Sports Med.

[2] Conlan T, Garth WP, Lemons JE (1998). Evaluation of the medial soft-tissue restraints of the extensor mechanism of the knee. J Bone Jt Surg Am.

[3] Desio SM, Burks RT, Bachus KN (1998). Soft tissue restraints to lateral patellar translation in the human knee. Am J Sports Med.

[4] Hautamaa PV, Fithian DC, Kaufman KR, Daniel DM, Pohlmeyer AM (1998). Medial soft tissue restraints in lateral patellar instability and repair. Clin Orthop Relat Res.

[5] Sinikumpu J, Nicolaou N (2023). Current concepts in the treatment of first-time patella dislocation in children and adolescents. J Child Orthop.

[6] Bailey MEA, Metcalfe A, Hing CB, Eldridge J, BASK Patellofemoral Working Group (2021). Consensus guidelines for management of patellofemoral instability. Knee.

[7] Elias JJ, Cosgarea AJ (2006). Technical errors during medial patellofemoral ligament reconstruction could overload medial patellofemoral cartilage: a computational analysis. Am J Sports Med.

[8] Stephen JM, Lumpaopong P, Deehan DJ, Kader D, Amis AA (2012). The medial patellofemoral ligament: location of femoral attachment and length change patterns resulting from anatomic and nonanatomic attachments. Am J Sports Med.

[9] Maione A, Tradati D, Ferrua P, Ricci M, Usellini E, Randelli PS, Berruto M (2023). Accuracy of femoral tunnel positioning in medial patellofemoral ligament reconstruction: anatomic insertion leads to better clinical outcome. Knee Surg Sports Traumatol Arthrosc.

[10] Walker M, Maini L, Kay J, Siddiqui A, Almasri M, de Sa D (2022). Femoral tunnel malposition is the most common indication for revision medial patellofemoral ligament reconstruction with promising early outcomes following revision reconstruction: a systematic review. Knee Surg Sports Traumatol Arthrosc.

[11] Tscholl PM, Ernstbrunner L, Pedrazzoli L, Fucentese SF (2018). The relationship of femoral tunnel positioning in medial patellofemoral ligament reconstruction on clinical outcome and postoperative complications. Arthroscopy.

[12] Herschel R, Hasler A, Tscholl PM, Fucentese SF (2017). Visual-palpatory versus fluoroscopic intraoperative determination of the femoral entry point in medial patellofemoral ligament reconstruction. Knee Surg Sports Traumatol Arthrosc.

[13] Rammohan R, Kotwal RS, Chandratreya A (2016). Intraoperative localisation of Schottle’s point without fluoroscopy during medial patellofemoral ligament reconstruction. Ann R Coll Surg Engl.

[14] Wang HJ, Song YF, Yan X (2021). Using anatomic landmarks to locate Schöttle’s point was accurate without fluoroscopy during medial patellofemoral ligament reconstruction. Arthroscopy.

[15] Zhang X, Xie G, Zhang C, Fang Z, Zhao J, Huangfu X (2019). Comparison and evaluation of the accuracy of the sulcus localization method to establish the medial patellofemoral ligament femoral tunnel: a cadaveric and clinical study. BMC Musculoskelet Disord.

[16] Schöttle PB, Schmeling A, Rosenstiel N, Weiler A (2007). Radiographic landmarks for femoral tunnel placement in medial patellofemoral ligament reconstruction. Am J Sports Med.

[17] Balcarek P, Walde TA (2015). Accuracy of femoral tunnel placement in medial patellofemoral ligament reconstruction: the effect of a nearly true-lateral fluoroscopic view. Am J Sports Med.

[18] Redfern J, Kamath G, Burks R (2010). Anatomical confirmation of the use of radiographic landmarks in medial patellofemoral ligament reconstruction. Am J Sports Med.

[19] Barnett AJ, Howells NR, Burston BJ, Ansari A, Clark D, Eldridge JD (2012). Radiographic landmarks for tunnel placement in reconstruction of the medial patellofemoral ligament. Knee Surg Sports Traumatol Arthrosc.

[20] Wijdicks CA, Griffith CJ, LaPrade RF, Johansen S, Sunderland A, Arendt EA, Engebretsen L (2009). Radiographic identification of the primary medial knee structures. J Bone Jt Surg Am.

[21] Kaipel M, Schützenberger S, Farr S, Gergely I, Vlcek A, Kainberger F, Boszotta H, Pretterklieber M (2015). Reliability of radiographic landmarks in medial patello-femoral ligament reconstruction in relation to the anatomical femoral torsion. Int Orthop.

[22] Chen J, Han K, Jiang J, Huangfu X, Zhao S, Zhao J, Xie G (2021). Radiographic reference points do not ensure anatomic femoral fixation sites in medial patellofemoral ligament reconstruction: a quantified anatomic localization method based on the saddle sulcus. Am J Sports Med.

[23] Chen J, Xiong Y, Han K, Xu C, Cai J, Wu C, Ye Z, Zhao J, Xie G (2022). Computed tomography imaging analysis of the MPFL femoral footprint morphology and the saddle sulcus: evaluation of 1094 knees. Orthop J Sports Med.

[24] Servien E, Fritsch B, Lustig S (2011). In vivo positioning analysis of medial patellofemoral ligament reconstruction. Am J Sports Med.

[25] Bobak CA, Barr PJ, O'Malley AJ (2018). Estimation of an inter-rater intra-class correlation coefficient that overcomes common assumption violations in the assessment of health measurement scales. BMC Med Res Methodol.

[26] Aframian A, Smith TO, Tennent TD, Cobb JP, Hing CB (2017). Origin and insertion of the medial patellofemoral ligament: a systematic review of anatomy. Knee Surg Sports Traumatol Arthrosc.

[27] Placella G, Tei M, Sebastiani E, Speziali A, Antinolfi P, Delcogliano M, Georgoulis A, Cerulli G (2015). Anatomy of the medial patello-femoral ligament: a systematic review of the last 20 years literature. Musculoskelet Surg.

[28] Dandu N, Trasolini NA, Hevesi M, Zavras AG, Elias TJ, Haneberg EC, Yanke AB (2022). Landmarks used in medial patellofemoral ligament reconstruction have variable topography. Arthrosc Sports Med Rehabil.

[29] Zaffagnini S, Dejour D, Grassi A, Bonanzinga T, Marcheggiani Muccioli GM, Colle F, Raggi F, Benzi A, Marcacci M (2013). Patellofemoral anatomy and biomechanics: current concepts. Joints.

[30] Reider B, Marshall JL, Koslin B, Ring B, Girgis FG (1981). The anterior aspect of the knee joint. J Bone Jt Surg Am.

[31] Huston KL, Okoroafor UC, Kaar SG, Wentt CL, Saluan P, Farrow LD (2017). Evaluation of the Schöttle technique in the pediatric knee. Orthop J Sports Med.

[32] Izadpanah K, Meine H, Kubosch J, Lang G, Fuchs A, Maier D, Ogon P, Südkamp NP, Feucht MJ (2020). Fluoroscopic guided tunnel placement during medial patellofemoral ligament reconstruction is not accurate in patients with severe trochlear dysplasia. Knee Surg Sports Traumatol Arthrosc.

[33] Sanchis-Alfonso V, Ramirez-Fuentes C, Montesinos-Berry E, Aparisi-Rodriguez F, Martí-Bonmatí L (2016). Does radiographic location ensure precise anatomic location of the femoral fixation site in medial patellofemoral ligament surgery?. Knee Surg Sports Traumatol Arthrosc.

[34] Siebold R, Borbon CA (2012). Arthroscopic extraarticular reconstruction of the medial patellofemoral ligament with gracilis tendon autograft-surgical technique. Knee Surg Sports Traumatol Arthrosc.

[35] Baldwin JL (2009). The anatomy of the medial patellofemoral ligament. Am J Sports Med.

[36] Nomura E, Horiuchi Y, Kihara M (2000). Medial patellofemoral ligament restraint in lateral patellar translation and reconstruction. Knee.

[37] Nomura E, Inoue M, Osada N (2005). Anatomical analysis of the medial patellofemoral ligament of the knee, especially the femoral attachment. Knee Surg Sports Traumatol Arthrosc.

[38] Ziegler CG, Fulkerson JP, Edgar C (2016). Radiographic reference points are inaccurate with and without a true lateral radiograph: the importance of anatomy in medial patellofemoral ligament reconstruction. Am J Sports Med.

[39] de Abreu-E-Silva GM, Buarque FAR, Dias TS, Lei P, Bueno ELR, de Andrade MAP (2020). Anatomical femoral tunnel positioning in the medial patellofemoral ligament reconstruction: is the free-hand technique accurate?. Ann Transl Med.

[40] Koh JL, Zimmerman T (2017). “Pin the tail on the MPFL” identification by palpation—results. Orthop J Sports Med.

[41] Heindel K, Smoak J, Kocan J (2023). Stiffness and instability after MPFL reconstruction using a fluoroscopic versus open technique to localize the femoral attachment site: a systematic review and meta-analysis. Orthop J Sports Med.

